# Unpacking externalising problems: negative parenting associations for conduct problems and irritability

**DOI:** 10.1192/bjpo.bp.115.000125

**Published:** 2015-07-20

**Authors:** Bonamy R. Oliver

**Affiliations:** **Bonamy R. Oliver**, PhD, School of Psychology, University of Sussex, Brighton BN1 9QH, UK. E-mail: bonamy.oliver@sussex.ac.uk

## Abstract

**Background:**

Reciprocal associations between negative parenting and child externalising problems are well documented, but measures commonly include child irritability, masking potential distinct associations for irritability and conduct problems.

**Aims:**

To illuminate links between negative parenting, child conduct problems and irritability over time.

**Method:**

A cross-lagged monozygotic (MZ) twin differences design was used in a UK sample (3154 twin pairs) at 4, 7 and 9 years.

**Results:**

Within-pair MZ differences in negative parenting were found to relate longitudinally to differences in conduct problems and irritability. Of note, negative parenting at age 7 was found to relate particularly to increased irritability at 9 years.

**Conclusions:**

Once genetics are taken into account, irritability in middle childhood may be particularly vulnerable to negative parenting, suggesting support for its malleability to parent-based intervention.

**Declaration of interest:**

None.

**Copyright and usage:**

© The Royal College of Psychiatrists 2015. This is an open access article distributed under the terms of the Creative Commons Attribution (CC BY) licence.

Numerous studies evidence a bidirectional relationship between negative parenting and child externalising behaviour. Commonly, such studies operationalise these externalising problems as encompassing both conduct problems (e.g. fighting, disobedience) and anger-prone behaviours such as temper tantrums and irritability. While negative parenting is associated with conduct problems from early in development, evidence suggests that parenting characterised by harshness and hostility can work specifically to increase a child's anger and irritability over time.^1^ In addition, irritability and other externalising problems have been shown to have critical, yet distinct, parts to play in short- and long-term adverse mental health outcomes,^2,3^ such that disentangling irritability from other externalising problems in the investigation of oppositional-defiant disorder symptoms is increasingly of interest clinically^4^ and in population samples.^5^ Thus, it is possible that previously found links between negative parenting and the development of externalising problems mask important distinctions in the longitudinal mechanisms involved in these two aspects of externalising behaviour. The current study is the first to make the distinction between conduct problems and child irritability in the same sample within the context of a longitudinal monozygotic (MZ) differences design.

The MZ differences method exploits the fact that MZ twins are genetically identical for inherited DNA sequence variation, such that differences between them can be attributed to non-shared environmental factors. The design is a ‘sharp scalpel’ for illuminating non-shared environmental influences – those that are specific to children in the same family and are associated with behavioural outcomes independent of genetically mediated mechanisms, including gene–environment correlation.^6^ Longitudinal MZ differences studies have demonstrated various aspects of parenting and the parent–child relationship to be associated with diverse child externalising behaviour problems over time, even after accounting for genetic confounds.^7,8,9,10^ The aim of the current study was to use a large UK sample to explore non-shared environmental links between negative parenting and child externalising behaviour. Specifically, a longitudinal cross-lagged panel design was used to examine within-pair MZ differences in child conduct problems and child irritability in association with MZ differences in negative parenting.

## Method

### Sample and procedure

The sampling frame for the current study was the Twins Early Development Study (TEDS), a population-based, longitudinal study of twins recruited from UK birth records and born in England and Wales in 1994–1996. TEDS has been demonstrated to be reasonably representative of the UK population and is described in detail elsewhere.^11^ Data at all ages were collected from the 1994 and 1995 cohorts of the study at 4, 7 and 9 years through postal questionnaires sent to parents. After medical exclusions, 3154 MZ twin pairs (52.9% girl–girl pairs) were included for whom parent data were available. Zygosity was assigned using DNA testing. The mean age of the sample when parent questionnaires were returned was 4.04 (s.d.=0.13), 7.07 (s.d.=0.25) and 9.02 (s.d.=0.29) years for the 4-, 7- and 9-year assessments respectively. Informed consent was obtained. TEDS was approved by the Institute of Psychiatry, King's College London Ethics Committee.

### Measures

#### Child conduct problems

Parent reports of child conduct problems were collected using the Strengths and Difficulties Questionnaire (SDQ),^12^ designed to measure behaviour problems and competencies in children aged 3–16 years. The conduct problem sub-scale comprises five items, of which four were used because the fifth, ‘child has temper tantrums’ refers to irritability and is included as such for the current study. These four items relate to stealing, lying, aggression and obedience (reverse coded) over the previous 6 months and are rated by parents as ‘not true’ (0), ‘somewhat true’ (1) or ‘certainly true’ (2). The SDQ's validity and reliability is well established in its standard form; reliabilities in this study reflected the diversity of behaviours described by the reduced number of items used in the scale (*α*=0.45, 0.51, 0.51 at ages 4, 7 and 9, respectively).

#### Child irritability

Two items relating to child irritability at ages 4 (child has temper tantrums, child is irritable) and 7 (child has temper tantrums, child is angry when corrected) and three items at age 9 (child has temper tantrums, child is touchy or easily annoyed, child is angry and resentful) were used and rated by parents about behaviours over the previous 6 months. Responses were in the form ‘not true’ (0), ‘somewhat true’ (1) and ‘certainly true’ (2) and were summed (*α*=0.72, 0.57, 0.73 at ages 4, 7 and 9, respectively).

#### Negative parenting

Parent reports of negative parenting were assessed by questionnaire, including two items relating to negative control (shouting and smacking) from a short discipline questionnaire adapted from a semi-structured interview,^13^ and four items relating to negative parental feelings (impatience, anger and frustration with child, and wishing the child would leave the parent alone) from an adapted short form of the Parental Feelings Questionnaire.^14^ At age 4, items were rated on a five-point scale (‘never’ to ‘usually’ for control and ‘untrue’ to ‘true’ for feelings) for first-born twins, and rated on a differential, five-point scale for second-born twins (‘a lot more’ to ‘a lot less’). Ratings for first-born twins were summed and standardised to a mean of zero and standard deviation of one for the TEDS sample. Ratings for second-born twins were then derived by standardising the differential score and adding it to the co-twin's standardised score. At ages 7 and 9 years, items were rated for each child as rarely/never (0), sometimes (1) and often (2). Items were summed and averaged by the number of control/feelings items in order to account for differences in the number of items in each domain (4 years: *α*=0.79; 7 years *α*=0.76; 9 years: *α*=0.74).

### Analyses

The MZ differences method capitalises on the fact that MZ twins growing up in the same family are genetically identical for inherited DNA sequence variation, such that differences between them can be attributed to non-shared environmental influences. These influences are free of shared experience, genetic influence and gene–environment correlation (the elicitation or shaping of experience due to genetic propensity), and can include gene expression, de novo mutations, epigenetic processes, intra- and extra-uterine environment and measurement error.^15^ To the extent that MZ differences in experience correlate with their differences in outcome, non-shared environmental effects are implicated.

Standardised residual scores controlled for age and for gender were used as is standard practice for twin studies, to ensure that twin correlations are not artificially inflated.^16^ Correlations among all measures used these residual scores for a random member of each twin pair only. Within-pair relative difference scores were calculated for negative parenting and child behaviour measures for all subsequent analyses. To account for birth order effects, these relative difference scores were generated by randomly assigning one member of each twin pair to be ‘Twin 1’ and the other to be ‘Twin 2’, and subtracting Twin 2 scores from Twin 1 scores on each measure. These difference scores were used in all longitudinal models for MZ twins only, with missing data accounted for using Full Information Maximum Likelihood in Mplus v 6.1.1.

Models estimated longitudinal, non-shared environmental pathways between MZ differences in negative parenting and MZ differences in child externalising behaviours. In particular, the models were designed to examine the extent to which reciprocal non-shared environmental relationships were evident between (1) negative parenting and conduct problems and (2) negative parenting and irritability. Note that non-significant paths in such models do not suggest a lack of phenotypic association, they demonstrate only that any phenotypic association is not significantly mediated by non-shared environmental effects. Model fit was assessed using root mean square error of approximation (RMSEA ≤0.08), comparative fit index (CFI ≥0.90) and Tucker-Lewis index (TLI ≥0.90). All pathways representing associations between child externalising behaviours and negative parenting were compared for conduct problems and irritability using bias-corrected bootstrapped 95% confidence intervals based on 10 000 samples.

## Results

### Preliminary analysis

Phenotypic correlations for all measures are shown in Table 1. All correlations were in the expected direction, significant at *P*<0.001 and small to moderate in magnitude, accounting for between 2% (negative parenting at 4 with conduct problems at 9) and 22% (conduct problems with irritability at 9 years) of the variance. In order to assess the extent to which MZ twins differ in their conduct problems, irritability and exposure to negative parenting, MZ twin intraclass correlations were calculated (also shown in Table 1, on the main diagonal, italicised). Consistent with other studies of this kind, MZ twin correlations for all measures were moderate to high (mean *r*=0.71).

**Table 1:** Phenotypic correlations between all measures and MZ intraclass correlations

	Age 4			Age 7			Age 9		
			
	Conduct problems	Irritability	Negative parenting	Conduct problems	Irritability	Negative parenting	Conduct problems	Irritability	Negative parenting
Age 4									
Conduct problems	*0.66*	0.41	0.27	0.41	0.25	0.29	0.31	0.32	0.24
Irritability		*0.47*	0.31	0.27	0.41	0.31	0.16	0.36	0.25
Negative parenting			*0.75*	0.22	0.24	0.49	0.15	0.25	0.43

Age 7									
Conduct problems				*0.62*	0.39	0.39	0.41	0.40	0.31
Irritability					*0.77*	0.40	0.25	0.46	0.29
Negative parenting						*0.71*	0.22	0.35	0.53

Age 9									
Conduct problems							*0.68*	0.47	0.37
Irritability								*0.81*	0.46
Negative parenting									*0.92*

A random member of the twin pair was selected to account for non-independence; all correlations are significant at *P*<0.001; italicised numbers on the main diagonals represent monozygotic (MZ) intraclass correlations.

### MZ differences analyses: negative parenting and child behaviour

**Fig. 1:**
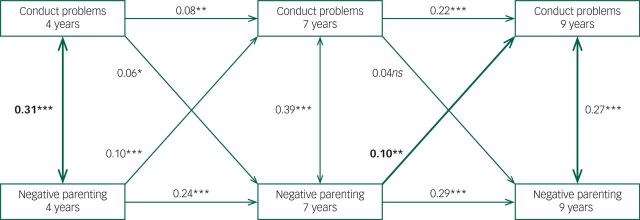
Cross-lagged model of monozygotic (MZ) differences in conduct problems with negative parenting at 4, 7 and 9 years. Note: Standardised coefficients are shown for within-time correlations (double-headed arrows) and autoregressive and cross-lagged path coefficients (single-headed arrows). Heavy weighted paths and associated coefficients in bold indicate externalising behaviour–parenting paths that differ significantly for conduct problems and irritability according to bias-corrected bootstrapped confidence, based on 10 000 samples; ***P<0.001, **P<0.01, *P<0.05.

Cross-sectional and longitudinal associations between within-pair MZ differences in negative parenting and child behaviour were modeled in two separate cross-lagged models, one for child conduct problems and one for child irritability. Models were found to fit satisfactorily for both conduct problems (*χ*^2^(4)=2.56, *P*=0.634; RMSEA=0.00 (90% CI 0.00–0.02); CFI=1.00; TLI=1.01) and for irritability (*χ*^2^(4)=11.07, *P*=0.026; RMSEA=0.02 (90% CI 0.01–0.04); CFI=0.99; TLI=0.97). The results of these cross-lagged models are shown in Fig. 1.

Within-time non-shared environmental associations between negative parenting and conduct problems (represented by double-headed arrows in Fig. 1) were evident, indicating that members of a twin pair reported by parents to experience more parental negativity than their co-twin were also reported to be the twin of the pair showing more conduct problems. Autoregressive pathways (that is, relationships within domain across ages 4, 7 and 9 years), suggested some non-shared environmental stability in conduct problems particularly in middle-late childhood, and in negative parenting over time. Of primary focus here are the longitudinal cross-trait connections given by the cross-lagged paths. These paths indicate the extent to which MZ differences in conduct problems and in negative parenting influence one another, accounting for within-trait stability. These cross-lagged path coefficients indicated some bidirectionality, at least in early childhood. That is, members of the twin pair who received more negative parenting than their co-twin at ages 4 and 7 had accordingly higher levels of conduct problems at ages 7 and 9, respectively, while those with higher levels of conduct problems at age 4 than their co-twin experienced more negative parenting at age 7. The cross-lagged path from conduct problems at age 7 to negative parenting at age 9 was not significant.

**Fig. 2:**
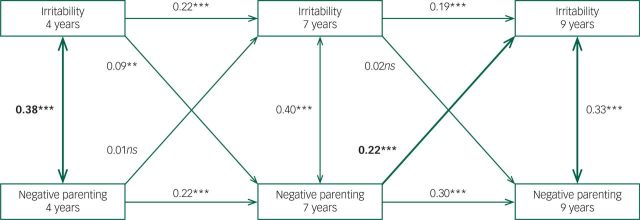
Cross-lagged model of monozygotic (MZ) differences in child irritability with negative parenting at 4, 7 and 9 years. Note: Standardised coefficients are shown for within-time correlations (double-headed arrows) and autoregressive and cross-lagged path coefficients (single-headed arrows). Heavy weighted paths and associated coefficients in bold indicate externalising behaviour–parenting paths that differ significantly for conduct problems and irritability according to bias-corrected bootstrapped confidence, based on 10 000 samples; ***P<0.001, **P<0.01, *P<0.05.

For irritability (see Fig. 2), within-time associations with negative parenting were also evident, demonstrating that twins of a pair reported to experience more parental negativity than their co-twin were also rated as showing more irritability than their twin. Autoregressive paths suggested some non-shared environmental stability of child irritability across age, similar from early to middle childhood and from middle to late childhood. Cross-lagged paths indicated no significant association between MZ differences in negative parenting at age 4 and irritability at age 7, rather, child irritability differences at age 4 were significantly associated with differences in negative parenting exposure at age 7; in other words, the member of the twin pair showing greater irritability at age 4 experienced more parental negativity at age 7 than their co-twin; this pattern did not continue in middle to late childhood. Twins showing more irritability than their co-twin at age 7 did not receive significantly more negative parenting than their co-twin at age 9; instead, twins reported to have more exposure to negative parenting than their co-twin at age 7 demonstrated considerably more irritability than their co-twin at age 9.

To assess statistically whether there were differences in the pathways for child conduct problems and irritability in non-shared environmental associations with negative parenting, bias-corrected bootstrapped confidence intervals based on 10 000 samples were examined. According to these data, the within-time correlations at age 4 and age 9 were significantly higher for irritability than for conduct problems, as was the non-shared link between negative parenting at age 7 and child irritability at age 9.

## Discussion

Capitalising on the powerful quasi-experimental MZ differences design in a large UK twin sample, the current study sought to elucidate non-shared environmental associations between negative parenting and two aspects of externalising behaviour: conduct problems and irritability. Non-shared environmental links with negative parenting were evident for both conduct problems and irritability, but negative parenting influences in middle childhood for later child irritability were of particular note. The results suggest that well-documented links between negative parenting and externalising problems may have non-shared environmental foundations in the more irritable, anger-prone aspects of these behaviours, and implicate key parenting influences in middle childhood for irritability, a trait likely to have long-term, adverse outcomes.^2,3^

The importance of unpicking irritability from other externalising problems has increasingly been highlighted. In psychiatric terms, there has been a conceptual change in understanding the role of irritability for mental health, not least evidenced by the recent addition of disruptive mood dysregulation disorder – characterised by severe temper tantrums and persistent irritability – to the Diagnostic and Statistical Manual of Mental Disorders.^4^ Similar growing recognition of this shift is evidenced in research, with examination of sub-categorisations of oppositional-defiant disorder symptoms – irritability and other externalising problems – including in population samples.^3,5^ However, longitudinal genetically informed studies examining non-shared environmental links between negative parenting and externalising problems have yet to follow this trend. Such studies have thus far concentrated on broader delineations that include irritability as one or two items on a more extensive externalising scale, thereby potentially masking important underlying mechanistic distinctions between different aspects of behaviour. Reconsideration of previously found non-shared environmental parenting associations for the development of externalising problems with this in mind would have considerable value, with a view to replicating the present findings across diverse samples and different developmental stages.

In the current study, after accounting for genetics, bidirectional effects between negative parenting and conduct problems were evident in the early years. For irritability, early pathways to negative parenting in middle childhood were significant, while those from negative parenting to irritability in middle childhood were not, congruent with findings elsewhere that child irritability may to some extent drive negative parenting in the early years.^17^ Nonetheless, as is typical in studies of this kind, these non-shared environmental connections early in development for both conduct problems and irritability were predominantly age-specific, and the magnitude of the paths was not large for either, nor were these early associations significantly different for the two aspects of behaviour. However, what is clearer from the current results, is that child irritability was particularly vulnerable to negative parenting over time, a finding that is in line with existing phenotypic studies.^1^ Indeed, the proportion of non-shared environmental variance in child irritability at age 9 accounted for by negative parenting at age 7 was almost 5%, a substantial proportion in the context of this conservative design. Alongside prior externalising behaviour connections that have shown stronger parental influences early in development – posited to be linked to widening environmental influences in later childhood and increases in genetic influence through adolescence and adulthood^8^ – the current findings hint at potentially distinct developmental mechanisms revealed in untangling irritability from other externalising problems.

Importantly, although the magnitude of non-shared environmental connections seen in the current context for conduct problems is small, these results do not imply that the associations are themselves small, as demonstrated by the phenotypic correlations which show moderate relationships. Instead, they point to probable genetic and shared environmental contributions to these relationships. A possible interpretation is that negative parenting associations with conduct problems are predominantly due to shared environmental influences and mutual genetic propensities to behaviours, as well as gene–environment interplay, where irritability in middle–late childhood has a stronger non-shared environmental contribution. Given long-term links between irritability and internalising problems specifically, this position is supported by suggestions that shared environmental influences may be more important for externalising problems, while differential experiences are key for internalising problems.^18^

As well as documenting early non-shared environmental connections between externalising problems and negative parenting, previous MZ twin differences studies have found amplified associations as a function of more extreme twin differences in negative parenting, proposing that more severe adverse experiences may have a greater impact on child outcomes.^8,19^ Of relevance, these studies have also shown increased associations for more extreme twin discordance in externalising problems, and have suggested that non-shared environmental influences may be diminished in unselected samples.^8^ In the current study, the identified non-shared environmental influences in middle childhood are compelling because of their distinct potency for irritability, and provide an additional – albeit speculative – explanation. In population-based samples, irritability and anger-proneness may be more common than other externalising difficulties, a supposition endorsed by emerging findings from a recent survey of 18 000 UK families (A. Stringaris, personal communication, 2014). Accordingly, in these samples, selecting for the most marked twin differences in externalising problems may be a biased selection toward children more discordant for irritability, such that the stronger association with differences in negative parenting illuminated could be due to the type of behaviour rather than – or as well as – the extremity of the discordance. This conjecture warrants further investigation; if borne out, the implication is that adverse experiences may have greater impact in unselected samples when one considers more common aspects of externalising behaviours.

A further point worthy of discussion tentatively links the current findings to previously considered divisions between children with ‘hot’ and ‘cold’ conduct problems.^20^ Children with a ‘cold’ form of conduct problems are otherwise described as having callous–unemotional traits and low empathy, whereas those with ‘hot’ conduct problems are considered to have more explosive, irritable and anger-prone characteristics. These distinctions are important for many reasons, not least since there is some evidence that parenting-intervention response between these groups of children may differ, most likely due to differences in reward and punishment sensitivity.^21^ Findings in this area are mixed, however, and the current findings suggest that accounting for child irritability itself may shed some light on these anomalies. Indeed, a recent parenting-intervention study indicated that, despite equal improvements in received parenting, children with irritable (dysregulated) oppositional defiant symptoms improved in conduct problems more than children with headstrong behaviours.^22^

### Limitations and future directions

The current study has a number of strengths, not least its sample size and longitudinal nature. Most notably, the MZ differences design ensures interpretation of findings as non-shared environment explanations, untainted by genetic influence or gene–environment correlations. Naturally, care is advised in generalising to other non-twin samples, since the MZ twin relationship has unique characteristics – not least age and gender similarities – that may be important for understanding differential treatment;^23^ however, research in other types of sibling pairs linking differential treatment with child behaviour mitigates many concerns.^24^ Since the measures used here are brief, and not specifically designed for the question in hand, additional research with more detailed measures would be of interest, as would an observational approach in order to reduce the potential for rater bias that may have reduced or increased reported within-pair differential treatment and child behaviours. However, while non-shared environmental stability within traits was modest, such measurement flaws are unlikely to account for the pattern of these findings. The phenotypic consistency of the measures adds confidence to their reliability, with genetic or shared environmental explanations for stability probable. Finally, since irritability and conduct problems are related, accounting for their covariance in these models is of interest. Indeed, initial models included both aspects of externalising problems together; however, the results remained largely unaltered such that, for clarity, the results are presented separately here.

The first of its kind, the present study requires replication and extension, including the examination of gender differences as well as plausible indirect pathways – beyond the scope of the current paper – between conduct problems, irritability and negative parenting. Here, important distinctions in the mechanisms of change for irritability and conduct problems are highlighted. Within this framework, unpacking known links between negative parenting and parental psychopathology would be of interest, since recent findings show associations between irritability and parental psychopathology, and stress the significance of parenting and of genetic and environmental mechanisms through which these links are manifest.^25^ Furthermore, parents are but one influence; teasing out irritability and conduct problems in the consideration of other non-shared experiences important for externalising problems, such as peer relations,^26^ would be of great interest.

For some time, child problem behaviour has been known to have important homotypic and heterotypic continuity. As we uncover potential specificity in child behaviour predictions of long-term mental health outcomes, unpicking parallel specificity in parenting associations may hold clues to better understanding developmental mechanisms responsible for development, and, ultimately, knowing how to intervene.
